# Supplementary material for: Analysis and Interpretation of Automated Blood Count in the Treatment of Chronic Paracoccidioidomycosis
Source: J Fungi (Basel). 2024 Apr 27;10(5):317. doi: 10.3390/jof10050317 (PMC11122400; doi:10.3390/jof10050317)
Supplement: Supplementary file 1 [file jof-10-00317-s001.zip › jof-2931275-supplementary.pdf]

Supplementary information 1 (Table S1). Hematological parameters analyzed and reference normal values.

Hematological parameters	Reference values
**Red series parameters ***	
Total erythrocyte count	
Males	4.5 to 6.5 million/mm³
Females	3.9 to 5.6 million/mm³
Hemoglobin (Hb)	
Males	13.5 to 17.5 g/dL
Females	11.5 to 15.5 g/dL
Hematocrit (Hct)	
Males	40 to 52%
Females	36 to 48%
Mean hemoglobin corpuscular volume (MCV)	80 to 95 fL
Mean hemoglobin concentration (MCH)	27 to 34 pg
Mean cell hemoglobin concentration (MCHM)	30 to 35 g/dL
**White series parameters ***	
Total leukocyte count	4,000 to 11,000/mm³
Neutrophils	1,800 to 7,500/mm³
Lymphocytes	1,500 to 3,500/mm³
Monocytes	200 to 880/mm³
Eosinophils	40 to 440/mm³
Basophils	10 to 100/mm³
**Platelet parameter**	150 to 400 x10^3/mm³
**Cell immaturity parameters #**	
Immature granulocytes (IG)%; (IG)	0 to 0.6%; 0 to 0.06 x10^3/µL
Reticulocytes (Ret)%	0.5 to 2.5%
Reticulocyte hemoglobin content (Ret-He)	28 to 36 pg
Fraction of immature reticulocytes (IRF)	1.6 to 10.5%

Source: ***[19]Hoffbrand and Steensma. 2020 #** [20] **Pekelharing et al., 2010**

Supplementary information 2 (Table S2). Evaluation of the blood red cells, white blood cells count (in number per cubic millimeter) and platelets count before treatment and its progress after introduction of the antifungal treatment.

Variable	Patients (number)	S0 Median [Q1;Q3]	S1 Median [Q1;Q3]	S2 Median [Q1;Q3]	S3 Median [Q1;Q3]	*p* value
**Red blood series**						
Erythrocytes	55	4.7 [4.3; 5.1]	...	4.8 [4.5; 5.4]	...	**0.001**
Erythrocytes	29	4.7 [4.4; 5.1]	4.7 [4.4; 5.2]	4.8 [4.5; 5.1]	4.8 [4.4; 5.3]	0.196
Hemoglobin	55	13.7 [12.1; 14.9]	...	14.6 [13.6; 15.6]	...	**<0.001**
Hemoglobin	29	13.9 [13.2; 14.8] [cd]	14.1 [13.5; 15.3] [bc]	14.6 [13.9; 15.2] [b]	15.1 [14.0; 16.1] [a]	**<0.001**
Hematocrit	55	42.1 [36.8; 45.6]	...	44.2 [40.5; 47.2]	...	**<0.001**
Hematocrit	29	42.2 [38.6; 45.0] [bc]	42.5 [40.7; 44.7] [b]	44.3 [41.4; 45.7] [ab]	44.8 [41.5; 48.1] [a]	**0.009**
MCV	55	89.9 [86.7; 91.9]	...	90.0 [88.3; 94.1]	...	**0.004**
MCV	29	89.3 [87.0; 91.3] [c]	89.4 [86.6; 93.3] [c]	90.4 [88.8; 94.2] [ab]	90.9 [89.4; 94.5] [a]	**<0.001**
MHC	55	29.6 [28.0; 30.8]	...	30.3 [29.2; 31.6]	...	**<0.001**
MHC	29	29.6 [27.8; 31.3] [cb]	29.4 [28.5; 31.4] [bc]	30.3 [29.2; 31.7] [ab]	31.2 [29.7; 32.5] [a]	**0.003**

CMHC	55	33.1 [32.0; 33.8]	...	33.5 [32.6; 34.2]	...	**0.006**
CMHC	29	33.1 [32.2; 34.0]	33.4 [32.5; 33.8]	33.3 [32.7; 33.9]	33.8 [32.6; 34.4]	0.313
RDWsd	22	47.0 [44.0; 49.8]	...	46.3 [45.0; 48.0]	...	0.984
RDWsd	07	46.0 [44.0; 48.6]	49.0 [46.0; 53.0]	47.0 [45.8; 51.0]	46.0 [44.0; 50.5]	0.398
RDWcv	22	14.2 [13.6; 15.5]	...	14.0 [13.1; 14.8]	...	0.465
RDWcv	07	13.7 [13.3; 14.3]	14.2 [14.1; 15.1]	13.4 [13.1; 14.9]	13.7 [12.6; 14.3]	0.239
**White blood series**						
Leukocytes	55	8200 [7000; 10230]	...	7460 [6380; 8885]	...	**0.007**
Leukocytes	29	8150 [6910; 10260]	7970 [5930; 9820]	7640 [6410; 8700]	7570 [6480; 10080]	0.113
Neutrophils	55	5062 [4199; 6337]	...	4422 [3350; 5491]	...	**0.002**
Neutrophils	29	4821 [4215; 6102]	4303 [3683; 5355]	4422 [3305; 5366]	4350 [3318; 5734]	0.161
Eosinophils	55	222 [79.5; 448]	...	224 [130; 430]	...	0.897
Eosinophils	29	229 [72.7; 681]	357 [143; 565]	196 [147; 622]	234 [102; 455]	0.661
Basophils	55	0.00 [0.0; 59.1]	...	0.00 [0.0; 72.3]	...	0.071

Basophils	29	0.0 [0.0; 63.7]	0.0 [0.0; 63.0]	44.4 [0.0; 76.0]	0.0 [0.0; 64.0]	0.761
Lymphocytes	55	1997 [1493; 2664]	...	2197 [1665; 2640]	...	0.158
Lymphocytes	29	1956 [1582; 2548]	2135 [1648; 2630]	2113 [1654; 2653]	1995 [1544; 2554]	0.338
Monocytes	55	718 [525; 990]	...	548 [423; 787]	...	**<0.001**
Monocytes	29	718 [565; 953][a]	717 [367;829][ac]	549 [420; 771][bc]	550 [515; 813][c]	**0.004**
NRL	55	2.6 [1.8; 3.5]	...	2.0 [1.5; 3.2]	...	**0.004**
NRL	29	2.5 [1.6; 3.2]	2.1 [1.6; 2.6]	2.1 [1.5; 3.2]	2.1 [1.5; 3.0]	0.392
MLR	55	0.4 [0.2; 0.5]	...	0.3 [0.2; 0.4]	...	**<0.001**
MLR	29	0.39 [0.26; 0.48] [a]	0.31 [0.22; 0.41] [ab]	0.28 [0.21; 0.41][b]	0.31 [0.25; 0.40] [ab]	**0.042**
**Platelet series**						
Platelets	55	322 [259; 403]	...	263 [220; 303]	...	**<0.001**
PLR	55	0.2 [0.1; 0.3]	...	0.1 [0.1; 0.2]	...	**<0.001**
Platelets	29	304 [256; 402] [a]	268 [228; 306][bcd]	242 [211; 298] [cd]	258 [209; 295] [d]	**< 0.001**
PLR	29	0.2 [0.1-0.2][a]	0.1 [0.1-0.2][bcd]	0.1 [0.1-0.1][cd]	0.1 [0.1-0.1][d]	**0.002**

Results presented as; median [Q1: first quartile; Q3: third quartile]; MCV: mean hemoglobin corpuscular volume; MCH: mean hemoglobin concentration; CHCM: mean corpuscular hemoglobin concentration; RDWsd: range of distribution of erythrocytes - expressed as standard deviation; RDWcv: range of distribution of erythrocytes - expressed as coefficient of variation; NLR: neutrophil/lymphocyte ratio; MLR: monocyte/lymphocyte ratio; PLR: platelet/lymphocyte ratio; Platelet count: number $x10^3$/cubic millimeter; S**0** – before treatment; S**1** - between the beginning of treatment and after clinical improvement; S**2** - clinical cure; S**3** - serological cure.
Statistical tests: Wilcoxon W test or paired Student's T test; Friedman test – post hoc (Durbin-Conover). Lower-case letters compare medians in the same row; medians followed by different letters are statistically different ($p \leq 0.05$), while followed by the same letter or not followed by any letter do not differ ($p > 0.05$).

Supplementary information 3 (Table S3). Differences in values in hematological parameters between clinical cure (S2) and pre-treatment (S0), according to the antifungal used, in 55 patients with the chronic form of paracoccidioidomycosis.

**Parameters (cell/mm3)**	**Cotrimoxazole n=20**	**Itraconazole n= 35**	**p value Ŧ**
	**Median [Q1; Q3]**	**Median [Q1; Q3]**	
Erithrocytes*	0.015 [0.15; 0.63]	0.3 [-0.1; 0.46]	0.479
RetHe	2 [0.9; 4.4]	1 [0; -30.6]	0.569
Leukocytes	-2490 [-1080; -2940]	-340 [-1680; 590]	0.478
Neutrophils	-2262.2 [67.25; 25.95]	-3534.8 [-2270; -5667.2]	0.902
IG	0.05 [0.04; -0.05]	0.04 [-0.01-0.28]	0.870
Platelets **	-35 [-174; -54]	-36 [-21; -61]	0.700

*Erithrocytes: million/mm^3; Platelet count: number $x10^3/mm^3$.
Statistical tests: Wilcoxon signed rank test.
